# The Spirituality–Resilience–Happiness Triad: A High-Powered Model for Understanding University Student Well-Being

**DOI:** 10.3390/ejihpe15080158

**Published:** 2025-08-13

**Authors:** Moises David Reyes-Perez, Leticia Carreño Saucedo, María Julia Sanchez-Levano, Roxana Cabanillas-Palomino, Paola Fiorella Monje-Yovera, Johan Pablo Jaime-Rodríguez, Luz Angelica Atoche-Silva, Johannes Michael Alarcón-Bustíos, Antony Esmit Franco Fernández-Altamirano

**Affiliations:** 1 Facultad de Psicología, Universidad Nacional Mayor de San Marcos, Lima 00051, Peru; msanchezl@unmsm.edu.pe; 2 Facultad de Psicología, Universidad Autónoma del Estado de México, Ciudad de México 01000, Mexico; lcarrenos@uaemex.mx; 3 Escuela de Psicología, Universidad Tecnológica del Perú, Lima 00051, Peru; c26978@utp.edu.pe; 4 Escuela de Psicología, Universidad César Vallejo, Piura 14000, Peru; pmonjey@ucvvirtual.edu.pe (P.F.M.-Y.); jjaimero@ucvvirtual.edu.pe (J.P.J.-R.); abustiosj@ucvvirtual.edu.pe (J.M.A.-B.); 5 Facultad de Psicología, Universidad Nacional de Frontera, Sullana 20100, Peru; latoche@unf.edu.pe; 6 Facultad de Derercho, Universidad Señor de Sipán, Chiclayo 14000, Peru; altamiranoae@uss.edu.pe

**Keywords:** spirituality, resilience, happiness, higher education students, university well-being, educational psychology

## Abstract

This study examines the relationships between spirituality, resilience, and happiness among higher education students, exploring the moderating roles of religious belief and years of study based on developmental and religious coping theoretical frameworks. Developmental theory suggests that university students’ psychological resources evolve across academic years, while religious coping theory posits that individual differences in religious commitment may buffer spirituality’s protective effects on well-being outcomes. Using a quantitative cross-sectional approach, data were collected from 459 university students from environmental science programs across public and private universities in northern Peru. Participants were predominantly female (59.04%) and aged 18–24 years (73%). Three validated instruments were administered: the Personal Spirituality Scale, Connor–Davidson Brief Resilience Scale, and Subjective Happiness Scale. Religious beliefs were measured on a 5-point scale, while years of study was categorized by academic year. Results from partial least squares structural equation modeling revealed significant direct effects of spirituality on both happiness (β = 0.256, *p* < 0.001) and resilience (β = 0.274, *p* < 0.001), with resilience also significantly influencing happiness (β = 0.162, *p* < 0.05). The structural model demonstrated exceptional explanatory power, with spirituality explaining 97.1% of variance in resilience, while spirituality and resilience together accounted for 86.2% of variance in happiness. Contrary to theoretical expectations, neither religious beliefs (β = 0.032, *p* = 0.489) nor years of study (β = −0.047, *p* = 0.443) showed significant moderating effects. These results suggest that spirituality and resilience serve as universal contributors to student well-being, operating independently of specific religious orientations and academic progression. The findings support integrating spiritual development and resilience-building components into inclusive university student support programs.

## 1. Introduction

The psychological well-being of university students has emerged as a critical area of inquiry within educational psychology, with mounting evidence suggesting that spiritual development, psychological resilience, and subjective happiness constitute interconnected dimensions of student flourishing in higher education contexts. Contemporary research demonstrates that these psychological resources operate synergistically to support student adaptation during the challenging developmental period of university attendance, where individuals must navigate complex academic demands while simultaneously establishing personal identity and life direction (Schwalm et al., 2022; Roberto et al., 2020).

Spirituality, conceptualized as the individual search for meaning, purpose, and transcendence that encompasses but extends beyond formal religious practice, has been consistently linked to enhanced psychological well-being among university populations. This construct differs fundamentally from religiosity, which refers to organized religious behaviors, beliefs, and institutional affiliations, in that spirituality encompasses a broader spectrum of meaning-making processes, personal transcendence experiences, and values-based living that may occur within or outside traditional religious frameworks (Kuo et al., 2014; David et al., 2022). Research indicates that spirituality serves as a protective psychological resource that moderates the relationship between academic stress and mental health outcomes, with students who report higher spiritual engagement demonstrating greater capacity for managing university-related challenges (Güldaş & Karslı, 2023).

The relationship between spirituality and happiness in higher education contexts operates through multiple psychological mechanisms. Empirical evidence suggests that spiritual practices and beliefs enhance subjective well-being by providing frameworks for meaning-making during periods of uncertainty, fostering connection to transcendent values that buffer against academic pressures, and supporting the development of positive coping strategies (David et al., 2022; Carranza Esteban et al., 2021). However, the specific pathways through which spiritual development influences happiness among university students remain incompletely understood, particularly regarding the role of psychological resilience as a potential mediating mechanism.

Psychological resilience, defined as the capacity to adapt positively to adversity, recover from setbacks, and maintain psychological equilibrium under stress, represents another crucial determinant of student well-being in higher education. Contemporary research demonstrates that resilient students exhibit superior academic performance, reduced psychological distress, and enhanced life satisfaction compared to their less-resilient peers (Turner et al., 2017; López-Aguilar et al., 2023). The development of resilience appears particularly critical during the university years, when students face multiple developmental challenges including academic pressure, social adaptation, identity formation, and future career decision-making (Al Omari et al., 2023).

The interaction between spirituality and resilience in promoting student happiness represents a significant gap in the current educational psychology literature. While Zhang et al. (2011) (as cited in Sadeghifard et al., 2020) found that spiritual transcendence predicted positive outcomes including resilience, their findings revealed non-significant regression weights for certain relationships, highlighting the need for more nuanced investigation of these associations. Similarly, Sadeghifard et al. (2020) emphasized the necessity for improved understanding of how spirituality and resilience interact to impact well-being outcomes among higher education students, noting that existing research has inadequately addressed the complex interplay between these psychological resources.

Recent investigations have begun to illuminate the multifaceted nature of these relationships. Le et al. (2021) argued that spiritual and religious factors should be systematically addressed within coping and well-being research, particularly given their demonstrated capacity to buffer against acculturative stress and promote psychological adaptation. However, current research suffers from several critical limitations that impede comprehensive understanding of spirituality–resilience–happiness relationships in higher education contexts.

First, existing studies have typically examined these constructs in isolation or in pairwise relationships, failing to investigate their simultaneous interactions within integrated theoretical models. This fragmentary approach obscures the potential for complex mediation and moderation effects that may characterize real-world psychological functioning among university students. Second, there remains insufficient attention to individual difference factors that may influence the strength and direction of these relationships, particularly variables such as religious belief orientation and developmental stage within the university trajectory.

Third, geographical and cultural limitations characterize much of the existing research, with particularly notable gaps in Latin American contexts where spiritual and religious traditions may interact differently with educational experiences. The psychology of well-being in higher education has increasingly recognized the importance of examining adaptive psychological factors within specific cultural contexts, as universal models may inadequately capture the nuanced ways that spiritual resources operate across diverse student populations (Yadav et al., 2024; Pansini et al., 2024).

The developmental considerations inherent in university education add further complexity to understanding spirituality–resilience–happiness relationships. Students experience significant psychological changes across their university tenure, with first-year students facing unique adaptation challenges related to transition and identity formation, while upper-level students encounter different stressors related to career preparation and future planning (Sundarasen et al., 2024; Vera et al., 2024). These developmental differences may moderate the relationships among spirituality, resilience, and happiness, yet existing research has inadequately addressed how these psychological resources function across different stages of university experience.

Furthermore, the role of religious belief as a moderating factor requires systematic investigation. While spirituality and religiosity represent distinct constructs, individual differences in religious commitment and practice may influence how spiritual resources translate into psychological benefits. Some students may derive spiritual meaning through formal religious frameworks, while others may engage spiritual development through secular approaches to meaning-making and transcendence. Understanding how these different orientations influence the effectiveness of spiritual resources for promoting resilience and happiness represents a critical gap in current knowledge.

The present study addresses these limitations by investigating the complex interrelationships among spirituality, resilience, and happiness within a comprehensive theoretical model that examines both direct and moderated effects. The principal objective of this research is to examine the moderating roles of religious belief and years of study in the relationships between spirituality and resilience, spirituality and happiness, and resilience and happiness among university students. Specifically, this investigation pursues five key objectives: first, to determine the direct effect of spirituality on happiness in higher education students; second, to assess the direct influence of resilience on happiness among university populations; third, to examine how spirituality influences resilience development in higher education contexts; fourth, to evaluate whether religious belief significantly moderates the relationship between spirituality and happiness; and fifth, to investigate whether years of study moderates the effect between resilience and happiness in university settings.

This investigation is situated within the understudied context of Latin American higher education, focusing on environmental science students who represent a unique population facing both traditional academic challenges and contemporary concerns about global environmental sustainability. This research context provides an opportunity to examine spirituality–resilience–happiness relationships within a specific disciplinary framework where students must integrate technical knowledge with broader meaning-making processes related to environmental stewardship and global responsibility.

The theoretical significance of this research lies in its potential to advance understanding of how spiritual development and psychological resilience interact to promote student well-being, while simultaneously examining boundary conditions that may influence these relationships. From a practical perspective, the findings have implications for developing evidence-based interventions to support student success and well-being in higher education contexts, as recommended by Ross et al. (2016) and Prescott et al. (2024). The integration of spiritual considerations within higher education programming has been increasingly recognized as contributing to student well-being and learning sustainability, with institutions seeking empirically grounded approaches to holistic student development (Fuertes, 2024; Green, 2023). Understanding how spiritual resources, resilience capacities, and individual differences interact to influence student happiness provides essential foundational knowledge for designing comprehensive support programs that address the multifaceted needs of contemporary university populations (Wolfe, 2015; Codato et al., 2011).

## 2. Literature Review

### 2.1. Review of Spirituality, Resilience, and Happiness in Higher Education Students: Findings, Challenges, and Opportunities in Educational Psychology

Fundamental findings indicate that spirituality and social support have a significantly positive effect on resilience among higher education students, particularly in the final semesters (Bukhori et al., 2017). This effect is strengthened by the findings of David et al. (2022), who demonstrated that forgiveness and gratitude are positively associated with happiness and academic performance among university students, with spirituality moderating the effect between forgiveness and student happiness. Systematic research has shown that religiosity and spirituality significantly impact university students’ lifestyles, establishing a positive relationship with the reduction in issues such as depression and academic stress (Duche-Pérez et al., 2024).

However, the study of these constructs in educational psychology faces significant challenges. Brewer et al. (2019a) identified conceptual and methodological weaknesses in the contemporary resilience literature, which complicate the design of effective interventions for higher education. Moreover, there is a tendency to view resilience as an individual phenomenon without adequately accounting for sociocultural and educational factors that influence its development among university students (Brewer et al., 2019b). In terms of academic impact, Ononye et al. (2021) reported that academic resilience is positively related to emotional intelligence and academic performance in higher education, with emotional resilience mediating the effect between these factors. This interaction complements the findings of Hatami and Shekarchizadeh (2022), who revealed that spiritual health is associated with an increase in happiness among university students, with resilience mediating this effect.

Interventions to promote these aspects in higher education have also shown promising results. Goodchild et al. (2023) demonstrated that incorporating a resilience development module into a first-year university foundational course leads to significant increases in student resilience when facing academic challenges. However, as Abulfaraj et al. (2024) noted, a comprehensive approach to supporting higher education students is needed, emphasizing the importance of multifaceted strategies to improve mental health and well-being in the face of academic and personal challenges. These findings underscore the need to continue developing effective, holistic interventions that consider the interrelationship among spirituality, resilience, and happiness in higher education, particularly for university students who face complex developmental and academic challenges in their educational journeys.

### 2.2. Spirituality Influences Happiness in Higher Education Students

Studies have shown that university students with higher spirituality scores report better health and life satisfaction (Anand et al., 2015), which complements findings by Jalilian et al. (2017), who identified a significant, positive relationship between spiritual health and happiness in higher education students. Research has also revealed that spirituality functions as an essential psychological resource for managing academic stress, playing a significant role in shaping university students’ identity, values, and sense of purpose (Ekwonye et al., 2020). This effect is further supported by findings from David et al. (2022), who demonstrated that the influence between spirituality and happiness is mediated by forgiveness and gratitude in higher education contexts. Additionally, Leung and Pong (2021) reported that all domains of spiritual well-being are negatively associated with academic anxiety and psychological distress, explaining significant variance in university students’ depression, anxiety, and stress. The importance of spirituality in the holistic development of higher education students has been emphasized by Sterk Barrett (2023), who suggested that university faculty members should consider their role in promoting spiritual growth. These findings align with research by Hayati et al. (2016) and Duffy (2010), who documented variations in the impact of spirituality by gender and its relationship to personal values, highlighting the multifaceted nature of this influence within higher education. Consequently, we propose the following:

**Hypothesis** **1.** *Spirituality significantly influences the level of happiness associated with higher education.*

### 2.3. Resilience as a Determinant of Happiness in Higher Education Students

Studies have shown that resilience is closely linked to mental health, well-being, and university students’ academic engagement (Turner et al., 2017), whereas López-Aguilar et al. (2023) reported that higher resilience levels influence academic decisions and general well-being. This effect is strengthened by findings from Al Omari et al. (2023), who identified factors such as regular sleep, perceived academic stress, well-being, and self-esteem as significant predictors of positive resilience in higher education contexts. The Academic Resilience Model (ARM) proposed by Durso et al. (2021) explains how adaptability, self-control, personal organization, and academic awareness contribute to the development of resilience and, consequently, to university students’ well-being. Additionally, Abulfaraj et al. (2024) reported that mindfulness and education-focused interventions can reduce academic anxiety and stress, thereby improving well-being. The psychological mechanisms by which resilience affects happiness have been explained by Etherton et al. (2022), who proposed a model that includes academic self-efficacy, educational goals, and academic anxiety. These findings are supported by recent research from Cui et al. (2024) and Lin and Zhan (2024), which demonstrated that resilience mediates the effects among self-esteem, personality traits, and mental well-being and influences the relationship between educational strategies and student well-being. Therefore, we propose the following:

**Hypothesis** **2.** *Resilience significantly influences the level of happiness associated with higher education.*

### 2.4. Spirituality Influences the Resilience of Higher Education Students

This hypothesis is strongly supported by various studies documenting the significant influence of spirituality on resilience among university students. Studies have shown that university students use spiritual coping practices to manage academic challenges, highlighting spirituality as a significant psychological resource (Ekwonye et al., 2020). This effect is further supported by findings from Sadeghifard et al. (2020) and Anjum et al. (2024), who demonstrated a positive correlation between spirituality and resilience in higher education contexts, indicating that spirituality indirectly reduces academic anxiety and increases resilience. Research by Womble et al. (2013) and Nelms et al. (2007) confirmed the positive association between spirituality and academic resilience, whereas Bryant and Astin (2008) and Wortmann et al. (2012) explored how spiritual struggles can influence university students’ adjustment and resilience. Lee and Waters (2013) reported that spirituality is associated with lower levels of academic stress, and Kneipp et al. (2009) reported that both religiosity and spirituality significantly contribute to higher education adjustment. These findings are consistent with research by Nauli and Mulyono (2019) and Rani and Ghazi (2023), who reported correlations between levels of spirituality and academic resilience. Additionally, Bukhori et al. (2017) demonstrated that spirituality and social support significantly contribute to the resilience of final-year university students, whereas Philip et al. (2019) revealed the central role of religion/spirituality in enhancing psychological, cognitive, and academic functioning, suggesting its potential as a coping mechanism for educational challenges. Therefore, we propose the following:

**Hypothesis** **3.** *Spirituality significantly influences the resilience of higher education.*

### 2.5. The Mediating Role of Religious Belief and Years of Study in Higher Education

Hypotheses regarding the moderation of religious belief and years of study find partial support in the educational psychology literature. With respect to religious belief as a moderator, studies have shown that it positively influences university students’ happiness (Zuhdiyah et al., 2023), whereas higher spirituality levels are associated with better academic awareness and life satisfaction (Anand et al., 2015). This effect is further supported by findings from Nagel and Sgoutas-Emch (2007), who documented the connection between spirituality, personal beliefs, and academic behaviors among university students.

In terms of moderation by years of study, research reveals distinct patterns across academic progression stages in higher education. First-year students face unique challenges in developing psychological resources, with McGuinness and Nordstokke (2023) emphasizing the critical importance of mindful self-care and resilience development during this foundational period. Gilbertson et al. (2022) demonstrated that spirituality serves as a particularly crucial resource for first-year college students, finding that closeness to God was associated with greater well-being and belonging during this critical window for identity development. The transition to university life represents a particularly vulnerable stage where spirituality may serve as a protective factor against academic stress and adjustment difficulties, with spiritual struggles potentially affecting well-being outcomes during early college years (Gilbertson et al., 2022; Kneipp et al., 2009).

Contemporary research indicates that the academic year significantly impacts students’ stress responses and psychological adaptation. Barbayannis et al. (2022) reported that the academic year of study substantially affects academic stress levels among college students, with different years presenting unique developmental challenges. Howard et al. (2023) reported that spiritual resources demonstrate differential protective effects across academic years, suggesting that the relationship between spirituality and well-being may strengthen or weaken depending on students’ academic maturity and accumulated university experience. Additionally, a 2023 Harvard Graduate School of Education study revealed that 45% of college students experience hopelessness and anxiety, with students belonging to religious groups reporting significantly higher levels of life purpose and meaning (47%) than their atheist (34%) or agnostic (32%) peers (Mowreader, 2024).

Conversely, upper-level students demonstrate different psychological profiles, with Petrosyants (2017) reporting that academic resilience levels vary significantly by year of study, particularly noting that second-year students exhibit greater resilience than their first-year counterparts and senior students. This developmental trajectory suggests that the relationships among spirituality, resilience, and happiness may strengthen or weaken depending on students’ academic maturity and accumulated university experience. Bryant and Astin (2008) reported that spiritual struggles can differentially impact university students’ adjustment patterns across academic years, whereas Wortmann et al. (2012) reported that the relationship between spiritual coping and psychological outcomes varies according to students’ developmental stage within their university trajectory.

Research on senior students indicates that prolonged exposure to academic demands may either enhance or diminish the protective effects of spiritual resources. Newton (2023) conducted a qualitative narrative inquiry examining the intersectionality of spirituality and moral development among nine traditional-age college seniors and reported that spiritual development throughout the college years influences both academic and personal outcomes differently depending on students’ accumulated experiences and developmental stages.

These developmental considerations, combined with recent evidence from Chen et al. (2023) demonstrating that social support influences academic engagement through the mediating effects of life satisfaction and academic motivation, provide a robust theoretical foundation for exploring how both religious belief and years of study may influence the proposed relationships in higher education contexts. Although direct evidence on specific moderating effects remains limited, these findings collectively suggest that years of study may significantly moderate the relationships among spirituality, resilience, and happiness, with first-year students potentially showing stronger spiritual–happiness associations owing to greater reliance on meaning-making resources during transition periods, whereas upper-level students may demonstrate more complex interaction patterns influenced by accumulated academic and personal experiences. Consequently, we propose the following hypotheses:

**Hypothesis** **4.** *Religious belief significantly moderates the relationship between spirituality and happiness in higher education.*

**Hypothesis** **5.** *Years of study significantly moderates the effect between resilience and happiness in higher education.*

**Figure 1:**
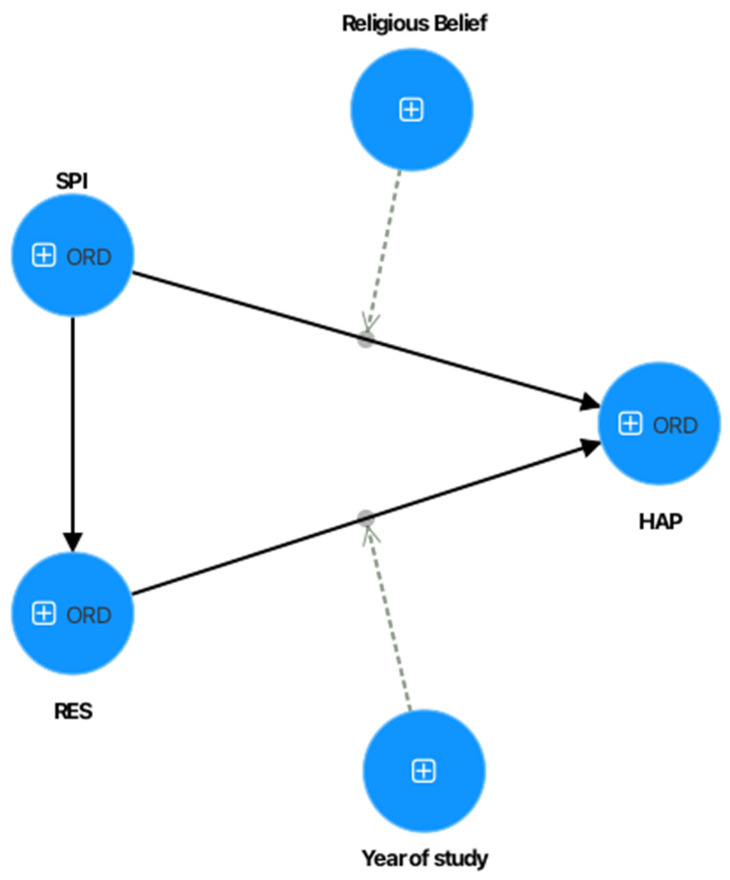
Proposed research model. Note: SPI = spirituality; RES = resilience; HAP = happiness.

Figure 1 presents the research model, which proposes the five hypotheses previously established.

## 3. Materials and Methods

To examine the research hypotheses, an empirical evaluation was conducted using a quantitative study with a correlational design and explanatory scope (Solórzano Solórzano et al., 2024). This methodology was selected to enable rigorous statistical analysis of the hypothesized relationships and their significance within the educational psychology framework, following established protocols for structural equation modeling in higher education research (Acosta et al., 2024; Hernández Sampieri & Mendoza Torres, 2018).

### 3.1. Participants

This study was conducted with 459 higher education students from environmental science and ecology programs at public and private universities in northern Peru. A non-probabilistic convenience sampling method was employed, which is methodologically appropriate for structural equation modeling studies examining psychological relationships in educational contexts where the research objective focuses on understanding patterns of association rather than population parameter estimation (Hair et al., 2017; Ringle et al., 2023). This sampling approach aligns with established practices in educational psychology research investigating spirituality, resilience, and well-being constructs, where theoretical model testing takes precedence over population generalizability (Kline, 2015).

The selection of environmental science and ecology students was strategically determined based on theoretical considerations relevant to spirituality–resilience–happiness relationships. Environmental science programs inherently integrate scientific knowledge with values-based decision-making processes, creating educational contexts where meaning-making, personal responsibility, and transcendent thinking are pedagogically emphasized. This population provides optimal conditions for examining spirituality constructs as these students regularly engage with questions of global responsibility, interconnectedness, and long-term consequence evaluation—cognitive processes that theoretically overlap with spiritual development frameworks (Ekwonye et al., 2020). Additionally, environmental science education emphasizes systems thinking and holistic approaches to problem-solving, which may facilitate the development of psychological resources such as resilience when facing complex, ambiguous challenges.

The sociodemographic composition of the sample reflects several important characteristics relevant to the study objectives. Gender distribution showed 59.04% female participants (271), 26.36% male participants (121), and 14.60% who preferred not to answer (67). This distribution is consistent with contemporary trends in STEM-related environmental programs, where female enrolment has increased substantially over the past decade. While gender differences in spirituality and well-being have been documented in previous research, the present study’s focus on examining structural relationships among constructs rather than group comparisons renders gender composition a descriptive rather than analytical concern for the primary research objectives.

Religious beliefs demonstrated heterogeneous distribution across the sample: 23.31% (107 participants) reported high religious belief levels (scores 4–5), 52.07% (239 participants) indicated moderate religious belief levels (score 3), and 24.62% (113 participants) expressed low religious belief levels (scores 1–2). This distribution provides adequate representation across different levels of religious commitment necessary for examining the proposed moderating effects while reflecting the diverse spiritual landscape characteristic of contemporary Peruvian higher education populations.

Academic year distribution revealed 34.86% (160 participants) in their first year, 25.27% (116 participants) in their third year, 12.64% (58 participants) in their second year, 7.62% (35 participants) in their fourth year, 3.49% (16 participants) in their sixth year, and 1.53% (7 participants) in their fifth year. This distribution enables examination of potential developmental changes across the university trajectory, which is essential for testing the proposed moderating effects of academic progression on spirituality–resilience–happiness relationships.

University and academic context characteristics further contextualize the higher education scope of this investigation. Participants were distributed across multiple faculties: 40.52% (186 participants) from Faculty of Engineering, 30.94% (142 participants) from Faculty of Sciences, 19.39% (89 participants) from Faculty of Natural Resources, and 9.15% (42 participants) from Faculty of Agriculture. The university-type distribution included 60.57% (278 participants) from public universities and 39.43% (181 participants) from private universities, reflecting the mixed higher education landscape in Peru.

Academic program diversity encompassed four main degree programs: Environmental Engineering (43.14%, 198 participants), Environmental Sciences (29.19%, 134 participants), Ecology and Natural Resources (18.95%, 87 participants), and Forestry Engineering (8.72%, 40 participants). Academic performance indicators showed a normal distribution, with 27.67% (127 participants) achieving high performance (GPA ≥ 16/20), 53.38% (245 participants) achieving medium performance (GPA 13–15.9/20), and 18.95% (87 participants) achieving lower performance (GPA < 13/20).

Socioeconomic and contextual factors provide additional insight into sample characteristics. Socioeconomic status distribution revealed 17.00% (78 participants) from high socioeconomic backgrounds, 64.92% (298 participants) from medium backgrounds, and 18.08% (83 participants) from low socioeconomic backgrounds. Place of residence indicated predominantly urban students (67.97%, 312 participants), with rural (19.39%, 89 participants) and semiurban (12.64%, 58 participants) representation. Employment status showed 62.96% (289 participants) as full-time students, 28.76% (132 participants) working part-time, and 8.28% (38 participants) working full-time. Complete sociodemographic and academic characteristics are presented in Table 1.

### 3.2. Instruments

Four validated instruments were administered for data collection:

Personal Spirituality Scale (SPI): The validated adaptation by González-Rivera et al. (2017) was employed to assess spiritual connection and practices through 12 items. This instrument specifically measures personal spiritual experiences, transcendent beliefs, and meaning-making processes distinct from organized religious practice. The scale includes items assessing connection to higher beings or forces, personal meditation and contemplative practices, values regarding respect for living beings, and experiences of universal connection. This operational definition addresses spirituality as a broader construct encompassing but extending beyond formal religious adherence, consistent with contemporary spirituality research frameworks. The instrument has demonstrated excellent psychometric properties in Latin American populations (α = 0.88).

Connor–Davidson Brief Resilience Scale (CD-RISC 10): The version validated by Bernaola Ugarte et al. (2022) for Peruvian university contexts was implemented. This instrument assesses adaptive capacity and recovery capabilities when facing adversity through 10 items measuring psychological flexibility, stress tolerance, and coping effectiveness. The scale has shown high internal consistency in university populations (α = 0.85).

Subjective Happiness Scale (SHS): The adaptation by Huamani Gomez and Mendoza Mori (2022) was utilized, comprising 4 items assessing subjective well-being and life satisfaction. This scale measures global happiness judgments and comparative well-being assessments, demonstrating adequate psychometric properties in Peruvian populations (α = 0.83).

Sociodemographic and Academic Variables: Religious beliefs were assessed using a single-item measure on a 5-point Likert scale (1 = “not at all religious” to 5 = “very religious”), following established practices in educational psychology research for measuring religious commitment (Pargament et al., 2011). Years of study was collected categorically by academic year to enable examination of developmental differences across university progression. Additional variables included age, gender, academic program, university type, academic performance, socioeconomic status, place of residence, and employment status.

All standardized instruments utilized 5-point Likert scales ranging from (1) “strongly disagree” to (5) “strongly agree.” Data collection employed a Google Forms digital questionnaire including informed consent, sociodemographic data, and the 26 evaluation items from validated scales.

### 3.3. Procedure and Data Analysis

Data collection occurred from June to September 2024 at environmental science departments of public and private universities in northern Peru. Following institutional approval, the instrument was distributed via institutional email and academic communication channels to eligible students. Participation was voluntary, with a detailed explanation provided of study objectives regarding educational psychology and student well-being research.

To address potential social desirability bias inherent in spirituality, happiness, and resilience assessment, several methodological safeguards were implemented. Anonymous data collection protocols were employed, with participants assured of confidentiality and research-only data usage. Instructions emphasized the importance of authentic responses for advancing understanding of student well-being, and reverse-coded items within scales provided internal consistency checks for response patterns.

The analytical process involved sequential stages designed for robust theoretical model testing:

Stage 1: Data preparation and descriptive analysis. Data cleaning was conducted using Microsoft Excel, followed by comprehensive descriptive analyses. Missing data patterns were evaluated systematically. Outliers were identified using standardized z-scores (|z| > 3.29) and visual inspection of boxplots, with six cases removed due to extreme values across multiple variables.
Stage 2: Measurement model validation. Confirmatory factor analysis assessed convergent and discriminant validity. Convergent validity was evaluated through factor loadings (threshold ≥ 0.70) and average variance extracted (AVE, threshold ≥ 0.50), following the recommendations of Hair et al. (2017). Items HAP5 and RES10 were removed due to insufficient factor loadings (<0.70), ensuring measurement model adherence to established psychometric criteria.
Stage 3: Reliability assessment. Internal consistency was evaluated using Cronbach’s alpha (α), composite reliability (CR), and rho_a coefficients, all exceeding the 0.70 threshold established by Hair et al. (2019).
Stage 4: Discriminant validity testing. Discriminant validity was assessed using the Fornell and Larcker (1981) criterion and heterotrait-monotrait (HTMT) ratios, with values below 0.85 indicating adequate discriminant validity (Ringle et al., 2023).
Stage 5: Structural model testing. Hypotheses were tested using partial least squares structural equation modeling (PLS-SEM) with SmartPLS v.4.0. PLS-SEM was selected over covariance-based SEM due to its appropriateness for predictive modeling, accommodation of smaller sample sizes, and flexibility with complex models including interaction effects. The structural model included direct effects (spirituality → happiness, resilience → happiness, spirituality → resilience) and moderating effects of religious belief and years of study.
Stage 6: Model evaluation. Model quality was assessed through coefficient of determination (R^2^), predictive relevance (Q^2^) using blindfolding procedures, effect sizes (f^2^), and model fit indices including standardized root mean square residual (SRMR), chi-square/degrees of freedom ratio (χ^2^/df), and normed fit index (NFI).

Statistical significance was evaluated using bias-corrected bootstrap confidence intervals (5000 bootstrap samples) with α = 0.05. Path coefficients were interpreted according to Cohen’s conventions: small (β ≈ 0.10), medium (β ≈ 0.30), and large (β ≈ 0.50). Moderation effects were tested through interaction terms with significant interactions examined via simple slope analysis.

Ethical considerations. All participants provided informed consent, and the study protocol received approval from the Ethics Committee of the Institute for Research, Innovation, Science and Technology (approval code 0128-2024-GM-IIICyT). The research adhered to Declaration of Helsinki principles, ensuring participant anonymity, voluntary participation, and withdrawal rights. Secure data storage protocols and confidential information handling were maintained throughout the study process.

## 4. Results

### 4.1. Results of the Measurement Model

In this research, partial least squares structural equation modeling (PLS-SEM) was used, which facilitated the performance of a confirmatory factor analysis (CFA) to validate the convergence of the measurement model. Table 2 shows the factor loadings of each item, which, according to the criteria of Hair (2009), reach values above 0.70, which is considered satisfactory. In addition, all the constructs analyzed present average variance extracted (AVE) values that exceed the threshold of 0.50, in accordance with the recommendations of Hair et al. (2017).

Table 3 presents the results of reliability and discriminant validity tests for the constructs. To assess reliability, Cronbach’s alpha coefficients (α) and composite reliability measures (CRs) (rho_a and rho_c) were calculated. According to the criteria established by Hair et al. (2019) and Acosta-Enriquez et al. (2024), values above 0.70 are considered adequate. Table 3 shows that all the constructs meet this standard, indicating adequate reliability in the measurements.

A multicollinearity test was performed using VIF, which showed that all constructs had values between 1 and 3, which is acceptable. Regarding the coefficient of determination (R^2^), the results indicate that the spirituality construct (SPI) explains 97.1% of the variance in resilience (RES), whereas spirituality (SPI) and resilience (RES) together account for 86.2% of the variance in happiness (HAP), indicating a high explanatory capacity for these constructs within the model.

For discriminant validity, Fornell and Larcker (1981) was applied, which establishes that the square root of the average variance extracted (AVE), represented by the diagonal values in the matrix, should be greater than the correlations between constructs located off the diagonal in the same row and column. As shown in Table 3, all the constructs meet this criterion, confirming discriminant validity. Additionally, the heterotrait-monotrait (HTMT) criterion was used, and the results indicate that all values are below the 0.85 threshold suggested by Ringle et al. (2023), further reinforcing the discriminant validity of the instrument used.

The goodness-of-fit indices presented in Table 4 are essential for assessing the convergent validity of the measurement model, providing researchers with a benchmark on the extent to which the obtained data align with theoretical values (Farhi et al., 2023; Hair et al., 2017).

The standardized root mean square residual (SRMR) is 0.073, which is below the recommended threshold of 0.85, according to Gaskin (2022), indicating an adequate model fit. Additionally, the chi-square over degrees of freedom ratio (χ^2^/df) is 1.763, which falls within the acceptable range of 1–3, as suggested by Escobedo Portillo et al. (2016), supporting the model’s fit acceptability. Finally, the normed fit index (NFI) is 0.998, surpassing the minimum threshold of 0.90 established by Escobedo Portillo et al. (2016), which confirms an excellent model fit

These results indicate that the measurement model has an adequate fit and meets the necessary criteria to be considered acceptable in terms of goodness of fit.

### 4.2. Testing the Research Hypotheses

**Figure 2:**
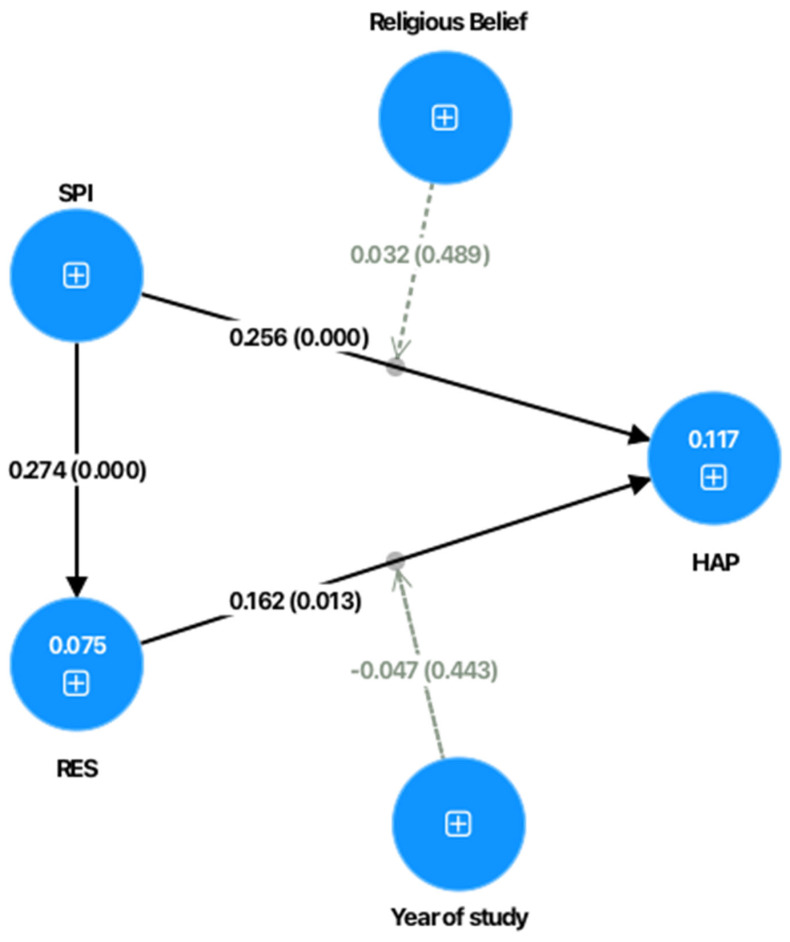
Resolved model.

Table 5 and Figure 2 show that, for Hypothesis 1 (H1), there is a significant direct effect of spirituality (SPI) on happiness (HAP), with a path coefficient of β = 0.256 *** and a *p* value of 0.000 ***. These findings indicate that spirituality directly influences the happiness of higher education students, demonstrating that spiritual practices, beliefs, and transcendent experiences serve as significant predictors of subjective well-being among university populations. The magnitude of this effect (β = 0.256) represents a medium-to-large effect size according to Cohen’s conventions, suggesting that spirituality constitutes a meaningful psychological resource for promoting happiness in higher education contexts.

Similarly, Hypothesis 2 (H2) reveals a significant direct effect of resilience (RES) on happiness (HAP), with a path coefficient of β = 0.162 ** and a *p* value of 0.013 **, suggesting that resilience also directly impacts happiness in higher education students. This finding indicates that students’ capacity for adaptation, recovery from adversity, and psychological flexibility significantly contribute to their overall subjective well-being and life satisfaction during their university experience. The effect size (β = 0.162) represents a small-to-medium effect, confirming that resilience is an important but complementary factor to spirituality in promoting student happiness.

Additionally, Hypothesis 3 (H3) confirms the significant positive effect of spirituality (SPI) on resilience (RES), with a path coefficient of β = 0.274 *** and a *p* value of 0.000 ***, indicating that spirituality contributes substantially to the development of resilience among higher education students. This relationship demonstrates that spiritual practices, meaning-making processes, and transcendent beliefs enhance students’ psychological capacity to cope with academic challenges, personal difficulties, and developmental transitions inherent in the university experience. The strong effect size (β = 0.274) suggests that spirituality may serve as a foundational resource that builds psychological resilience, which in turn contributes to overall well-being.

In contrast, Hypotheses 4 (H4) and 5 (H5), which proposed moderating effects of religious belief and years of study on the relationships among the constructs in higher education contexts, did not show significant effects, with p values above 0.05 (H4: β = 0.032, *p* = 0.489; H5: β = −0.047, *p* = 0.443), leading to the rejection of both hypotheses. The absence of significant moderation by religious belief (H4) suggests that the relationship between spirituality and happiness operates independently of specific religious orientations or denominational affiliations, indicating that the beneficial effects of spiritual practices and beliefs transcend particular religious frameworks among university students. Similarly, the lack of significant moderation by years of study (H5) indicates that the relationship between resilience and happiness remains stable across different stages of the university trajectory, from first-year students through senior-level students.

Table 6 presents the effect sizes, which are acceptable and strengthen the predictive ability of the suggested model. In addition, it strengthens the confirmation of the hypotheses put forward in the research.

## 5. Discussion

The results of this research provide significant empirical evidence on the interrelationships among spirituality, resilience, and happiness in higher education, as well as the moderating roles of religious beliefs and years of study. Analysis of the proposed hypotheses reveals important findings that contribute to understanding these constructs within the context of higher education and educational psychology, though these results warrant careful interpretation given methodological considerations.

With respect to the first hypothesis, the results confirm that spirituality significantly and positively influences the happiness of higher education students (β = 0.256, *p* < 0.001). This finding is consistent with those of previous studies by Anand et al. (2015) and Jalilian et al. (2017), who reported that university students with higher levels of spirituality reported better health and life satisfaction. The strength of this relationship supports Ekwonye et al.’s (2020) findings on the role of spirituality as an essential psychological resource for managing academic stress and for shaping identity and purpose in higher education settings.

For the second hypothesis, the results demonstrate that resilience has a significant effect on happiness (β = 0.162, *p* < 0.05), which aligns with research by Turner et al. (2017) and López-Aguilar et al. (2023) on the relationships among resilience, mental health, and student well-being in higher education. This finding supports the Academic Resilience Model (ARM) proposed by Durso et al. (2021), which explains how adaptability and self-control contribute to university students’ well-being.

The third hypothesis, positing a significant influence of spirituality on resilience, was also confirmed (β = 0.274, *p* < 0.001). This result is consistent with findings of Sadeghifard et al. (2020) and Anjum et al. (2024), who demonstrated a positive correlation between spirituality and resilience in higher education contexts. The strength of this relationship is reflected in the high coefficient of determination, with spirituality explaining 97.1% of the variance in resilience. While this represents a stronger association than typically reported in the educational psychology literature, it raises important theoretical questions about potential conceptual overlap between spirituality and resilience constructs, particularly given that spiritual practices often encompass coping mechanisms and meaning-making processes that may overlap with resilience dimensions.

A particularly noteworthy finding is the substantial explanatory power of the model, with spirituality and resilience together accounting for 86.2% of the variation in happiness among higher education students. These results exceed those reported in previous research, though this magnitude of explained variance may reflect the inherent limitations of cross-sectional self-report designs, including potential social desirability bias inherent in spirituality and happiness assessments, or method variance common to self-report measures administered simultaneously.

Contrary to the expectations outlined in the fourth and fifth hypotheses, no significant moderating effects of religious beliefs (β = 0.032, *p* > 0.05) or years of study (β = −0.047, *p* > 0.05) were found in the relationships studied. These results differ from the findings of Zuhdiyah et al. (2023) on the moderating role of religious tolerance and Petrosyants (2017) on variations in resilience by year of study in higher education. However, the absence of significant moderating effects requires cautious interpretation. The lack of statistical significance may reflect measurement limitations, insufficient statistical power for detecting interaction effects, or genuine absence of moderation. Alternative explanations include potential ceiling effects in the spirituality–happiness relationship or the possibility that our single-item religious belief measure may have lacked sufficient sensitivity to capture meaningful religious variations among participants.

The validity of these findings is strengthened by the robust psychometric indicators obtained, with model fit indices that meet established criteria (SRMR = 0.073, NFI = 0.998). The reliability and discriminant validity of the constructs, as evidenced by HTMT values below 0.85, suggest that the identified relationships represent meaningful patterns within the studied population. Nevertheless, several methodological considerations limit interpretative confidence. The convenience sampling approach and online recruitment may have introduced self-selection bias, potentially attracting participants with stronger spiritual orientations or well-being awareness. Additionally, the cross-sectional design precludes causal inferences, and the environmental science student sample may limit generalizability to broader university populations.

The implications of these findings should be considered within appropriate methodological constraints. While our results suggest potential value in incorporating spiritual development components into university student support programs, such recommendations require validation through longitudinal studies and randomized controlled trials before widespread implementation. The absence of significant moderating effects, rather than confirming universality, indicates the need for more nuanced measurement approaches and larger samples powered specifically for moderation analysis. Future research should also examine potential contextual factors, including university-type differences and cultural variations, that may influence these relationships in ways not captured by our current analysis.

### 5.1. Theoretical and Practical Implications

First, this research expands the theoretical understanding of the interrelationships among spirituality, resilience, and happiness in educational psychology, demonstrating that these constructs are more strongly linked in higher education students than previously documented in the literature. The model’s high explanatory power (R^2^ = 0.862 for happiness) suggests that the combination of spirituality and resilience may provide a more robust theoretical framework for understanding well-being in higher education than models that consider these factors in isolation.

Second, the absence of significant moderating effects of religious beliefs and years of study challenges previous conceptualizations of how these factors influence university students’ well-being. This finding suggests a need to reconsider existing theoretical frameworks in educational psychology that assume the universality of these moderating effects, instead proposing a more parsimonious model of the effects among spirituality, resilience, and happiness in higher education.

From a practical perspective, the results have direct implications for designing interventions in higher education settings. The strong influence of spirituality on both resilience and happiness suggests that university programs could benefit from integrating elements of spiritual development into their student support services. This might include creating spaces for contemplative reflection, mindfulness practices, and exploration of meaning and purpose within academic and career development programs.

Moreover, the finding that the effects of spirituality and resilience are not moderated by religious beliefs or years of study suggests that interventions based on these factors could be effective for diverse groups of higher education students. This has practical implications for designing more inclusive and universally applicable well-being programs in university settings.

### 5.2. Limitations and Future Research

First, the cross-sectional design of the study does not allow for definitive causal inferences among the variables studied in higher education contexts. Future studies could adopt longitudinal designs to examine how these relationships evolve over time during university experience and establish causal directionality with greater certainty.

Second, the sample was limited to students from environmental science programs in northern Peru, which may limit the generalizability of the findings to other academic disciplines and geographical contexts in higher education. Future research could benefit from including more diverse samples of university students across different academic fields and conducting cross-cultural comparisons to validate the universality of these relationships in various educational contexts.

A third limitation is the exclusive reliance on self-report measures, which may be subject to social desirability bias in educational settings. Future studies could incorporate mixed methods, including observational measures and qualitative interviews, to gain a deeper understanding of how spirituality and resilience influence happiness among university students.

Future studies could also explore other potential moderators not considered in this research, such as university type (public vs. private), academic discipline, socioeconomic factors, or cultural background. Additionally, it would be valuable to examine the specific mechanisms through which spirituality influences resilience and happiness in higher education contexts, possibly incorporating additional mediating variables such as academic self-efficacy, social support, a sense of belonging, or adaptive coping strategies.

Finally, given the model’s high explanatory power, future studies might investigate whether ceiling effects exist or whether other unconsidered factors could further enhance the understanding of well-being in higher education. It would also be valuable to examine whether these findings hold in contexts of academic crisis, institutional changes, or significant transitions within university settings.

The findings of this study contribute significantly to the growing field of educational psychology and provide valuable insights for enhancing the well-being of higher education students through the integration of spiritual and resilience-building practices in university programs.

## 6. Conclusions

This research significantly advances the understanding of the interrelationships among spirituality, resilience, and happiness in higher education students, as well as the potential moderating roles of religious beliefs and years of study. The findings reveal several novel contributions to the fields of educational psychology and student well-being research.

First, this study identifies an exceptionally strong effect between spirituality and resilience in higher education, with an explanatory power unprecedented in the educational psychology literature. These findings challenge previous conceptualizations that tended to underestimate the magnitude of this effect, suggesting that spirituality may be a more fundamental resource for the development of psychological resilience than previously considered in higher education research.

Second, the research shows that the combination of spirituality and resilience explains a substantial proportion of the variance in university students’ happiness, significantly exceeding the predictive levels reported in earlier studies of educational psychology. This finding suggests that these constructs may be central—rather than peripheral—elements in promoting well-being among higher education students.

A third significant finding is the absence of moderating effects from religious beliefs and years of study, which calls into question prevailing assumptions in the educational psychology literature regarding the universality of these moderating effects. This discovery suggests that the benefits of spirituality and resilience for well-being may be more universal among university students than previously thought.

The methodology employed in this study also represents a noteworthy contribution, particularly within the context of Latin American higher education research. The rigorous application of structural equation modeling techniques, combined with a comprehensive analysis of validity and reliability, establishes a robust methodological standard for future research in educational psychology and student well-being studies.

These findings have important implications for understanding how spiritual development and resilience building can enhance the psychological well-being of higher education students, ultimately contributing to more effective educational outcomes and student success. The strong interconnections identified between spirituality, resilience, and happiness suggest that incorporating these elements into university student support programs could significantly enhance the preparation of students for academic challenges and personal development throughout their educational journey.

From a practical standpoint, these results provide empirical support for university administrators, student affairs professionals, and educational psychologists to develop comprehensive well-being programs that integrate contemplative practices, resilience training, and meaning-making activities. The universal nature of these relationships across religious backgrounds and academic years suggests that such programs can be designed as inclusive initiatives that benefit diverse student populations in higher education settings.

The implications extend beyond individual student well-being to institutional effectiveness and educational quality. Universities that prioritize the development of spirituality and resilience among their students may see improvements in academic persistence, psychological health, life satisfaction, and overall educational outcomes. This holistic approach to student development aligns with contemporary trends in higher education that recognize the importance of addressing the whole person—intellectual, emotional, and spiritual dimensions—in the educational process.

Future research should continue to explore these relationships across different cultural contexts, academic disciplines, and institutional types to further validate and extend these findings. Additionally, the development and evaluation of specific interventions based on these findings will be crucial for translating this research into practical applications that can enhance the university experience and promote student flourishing in higher education environments.

## Figures and Tables

**Table 1:** Sociodemographic and academic characteristics of the sample (n = 459).

Feature	fi	%
Gender		
Female	271	59.04
Male	121	26.36
Prefer not to answer	67	14.60
Age group		
18–19 years old	168	36.60
20–24 years old	167	36.38
25–29 years old	26	5.66
30 or more years old	31	6.75
Missing data	67	14.60
Religious beliefs		
High (scores 4–5)	107	23.31
Moderate (score 3)	239	52.07
Low (scores 1–2)	113	24.62
Academic year		
First year	160	34.86
Second year	58	12.64
Third year	116	25.27
Fourth year	35	7.62
Fifth year	7	1.53
Sixth year	16	3.49
Missing data	67	14.60
Faculty/School		
Faculty of Engineering	186	40.52
Faculty of Sciences	142	30.94
Faculty of Natural Resources	89	19.39
Faculty of Agriculture	42	9.15
University type		
Public university	278	60.57
Private university	181	39.43
Degree program		
Environmental Engineering	198	43.14
Environmental Sciences	134	29.19
Ecology and Natural Resources	87	18.95
Forestry Engineering	40	8.72
Academic performance		
High (GPA ≥ 16/20)	127	27.67
Medium (GPA 13–15.9/20)	245	53.38
Low (GPA < 13/20)	87	18.95
Socioeconomic status		
High	78	17.00
Medium	298	64.92
Low	83	18.08
Place of residence		
Urban	312	67.97
Rural	89	19.39
Semiurban	58	12.64
Employment status		
Student only	289	62.96
Part-time work	132	28.76
Full-time work	38	8.28

Note: fi = Observed frequencies; GPA = Grade point average on a 0–20 scale (Peruvian higher education system); religious beliefs measured on a 5-point Likert scale (1 = not at all religious, 5 = very religious).

**Table 2:** Results of the confirmatory factor analysis (CFA).

Items		Factor Loading	Standard Deviation (STDEV)	*p* Values	AVE	Construct
In general, I consider myself: 1 = A not very happy person; 7 = A very happy person.	HAP1	0.722	0.043	0.000	0.594	Happiness (HAP)
Compared to most of my friends and/or colleagues, I consider myself to be 1 = Less Happy; 7 = Happiest	HAP2	0.735	0.064	0.000		
Some people are very happy. They enjoy life no matter what happens, they make the most of everything. To what extent does this characterization describe you? 1 = Not at all; 7 = A lot	HAP3	0.879	0.029	0.000		
Some people are very happy. Even if they have good reasons to feel sad, they can always be as happy as they want to be. To what extent does this description represent you? 1 = Not at all; 7 = A lot	HAP4	0.745	0.045	0.000		
I am able to adapt when changes arise.	RES1	0.769	0.060	0.000	0.652	Resilience (RES)
I am able to handle unpleasant/painful unpleasant/painful feelings such as sadness, fear and anger.	RES10	0.834	0.039	0.000		
I can cope with anything.	RES2	0.809	0.051	0.000		
When I face problems, I try to see the positive side of them.	RES3	0.778	0.033	0.000		
Facing difficulties can make me stronger.	RES4	0.889	0.048	0.000		
I tend to recover quickly after illness, injury or other difficulties.	RES5	0.805	0.037	0.000		
I believe I can achieve my goals, even if there are obstacles.	RES6	0.898	0.051	0.000		
Under pressure, I stay focused and think clearly.	RES7	0.843	0.053	0.000		
I am not easily discouraged by failure.	RES8	0.809	0.037	0.000		
I believe I am a strong person when faced with life challenges and difficulties.	RES9	0.709	0.036	0.000		
I believe in a higher being or force that provides me with support and sustenance in difficult times.	SPI1	0.854	0.056	0.000	0.754	Spirituality (SPI)
I feel a sense of connection and harmony with myself.	SPI10	0.898	0.027	0.000		
I have a personal relationship with an upper being or force.	SPI11	0.747	0.037	0.000		
Sometimes I feel connected to the universe.	SPI12	0.889	0.039	0.000		
I practice meditation to get in touch with myself.	SPI2	0.876	0.059	0.000		
Accepting and respecting the diversity of people is a value for me.	SPI3	0.954	0.074	0.000		
My faith in a higher being or force helps me face the challenges in my life.	SPI4	0.865	0.041	0.000		
I practice silence to get in touch with myself.	SPI5	0.850	0.052	0.000		
Maintaining and strengthening my relationships with others is important to me.	SPI6	0.734	0.073	0.000		
All living beings deserve respect.	SPI7	0.865	0.082	0.000		
Helping other people is a value for me.	SPI8	0.875	0.067	0.000		
I practice prayer to get in touch with an upper being or force.	SPI9	0.845	0.039	0.000		

**Table 3:** Reliability, discriminant validity, and coefficients of determination.

Construct	α	CR (rho_a)	CR (rho_c)	VIF	R^2^	Q^2^ Predict	HAP	RES	SPI	HTMT
HAP	0.779	0.789	0.876	1.673	0.862	0.899	0.776			0.402
RES	0.869	0.875	0.845	1.132	0.971	0.979	0.430	0.874		0.251
SPI	0.855	0.789	0.886	1.849	-	-	0.598	0.374	0.781	0.288

**Table 4:** Model fit.

Criteria	Estimated Model	Threshold	Author	Decision
SRMR	0.073	<0.85	(Gaskin, 2022)	Acceptable
d_ULS	2.250			
d_G	0.552			
χ^2^/df	1.763	Between 1 and 3	(Escobedo Portillo et al., 2016)	Acceptable
NFI	0.998	>0.90	(Escobedo Portillo et al., 2016)	Acceptable

**Table 5:** Results of hypothesis testing.

Hypothesis		*β*	*p* Value	Percentile		SD	Decision
				2.50%	97.50%		
H_2_	RES ⟶ HAP	0.162	0.013	0.044	0.299	0.065	Accepted
H_1_	SPI ⟶ HAP	0.256	0.000	0.160	0.357	0.051	Accepted
H_3_	SPI ⟶ RES	0.274	0.000	0.175	0.407	0.060	Accepted
H_5_	Religious Belief × RES ⟶ HAP	0.032	0.489	−0.057	0.125	0.047	Rejected
H_4_	Year of study × SPI ⟶ HAP	−0.047	0.443	−0.152	0.092	0.062	Rejected

Note. *β* = path coefficient.

**Table 6:** Effect size.

Construct	RES	SPI	HAP
HAP	0.789	0.865	-
RES	-	0.911	0.845
SPI	0.852	-	0.946

## Data Availability

The datasets used and/or analyzed during the current study are available from the corresponding author upon reasonable request.
